# Conservation and Divergence in Duplicated Fiber Coexpression Networks Accompanying Domestication of the Polyploid *Gossypium hirsutum* L

**DOI:** 10.1534/g3.120.401362

**Published:** 2020-06-25

**Authors:** Joseph P. Gallagher, Corrinne E. Grover, Guanjing Hu, Josef J. Jareczek, Jonathan F. Wendel

**Affiliations:** Ecology, Evolution, and Organismal Biology Dept., Iowa State University, Ames, IA, 50010

**Keywords:** cotton, domestication, gene coexpression, differential gene expression, fiber, polyploidy

## Abstract

*Gossypium hirsutum* L. (Upland cotton) has an evolutionary history involving inter-genomic hybridization, polyploidization, and subsequent domestication. We analyzed the developmental dynamics of the cotton fiber transcriptome accompanying domestication using gene coexpression networks for both joint and homoeologous networks. Remarkably, most genes exhibited expression for at least one homoeolog, confirming previous reports of widespread gene usage in cotton fibers. Most coexpression modules comprising the joint network are preserved in each subgenomic network and are enriched for similar biological processes, showing a general preservation of network modular structure for the two co-resident genomes in the polyploid. Interestingly, only one fifth of homoeologs co-occur in the same module when separated, despite similar modular structures between the joint and homoeologous networks. These results suggest that the genome-wide divergence between homoeologous genes is sufficient to separate their co-expression profiles at the intermodular level, despite conservation of intramodular relationships within each subgenome. Most modules exhibit D-homoeolog expression bias, although specific modules do exhibit A-homoeolog bias. Comparisons between wild and domesticated coexpression networks revealed a much tighter and denser network structure in domesticated fiber, as evidenced by its fewer modules, 13-fold increase in the number of development-related module member genes, and the poor preservation of the wild network topology. These results demonstrate the amazing complexity that underlies the domestication of cotton fiber.

Cotton (*Gossypium*) is the most important source of natural textile fibers globally. Among the four cultivated species, *G. hirsutum* L., also known as Upland cotton, is the most widely grown, and is responsible for more than 90% of cotton production worldwide. Wild *G. hirsutum*, native to coastal Yucatan, Mexico and more sparsely elsewhere in nearby regions (extending as far north as the Florida Keys), was domesticated approximately 5,000 years ago (Wendel *et al.* 1992; Brubaker and Wendel 1994; d’Eeckenbrugge and Lacape 2014). Following initial domestication in or around the Yucatan Peninsula, *G. hirsutum* spread rapidly throughout Central America, where semi-domesticated or “door-yard” forms are still found today. In the last several hundred years, strong directional selection for enhanced fiber and other agronomic traits led to the modern forms of Upland cotton, which are grown globally today (Figure 1).

**Figure 1:**
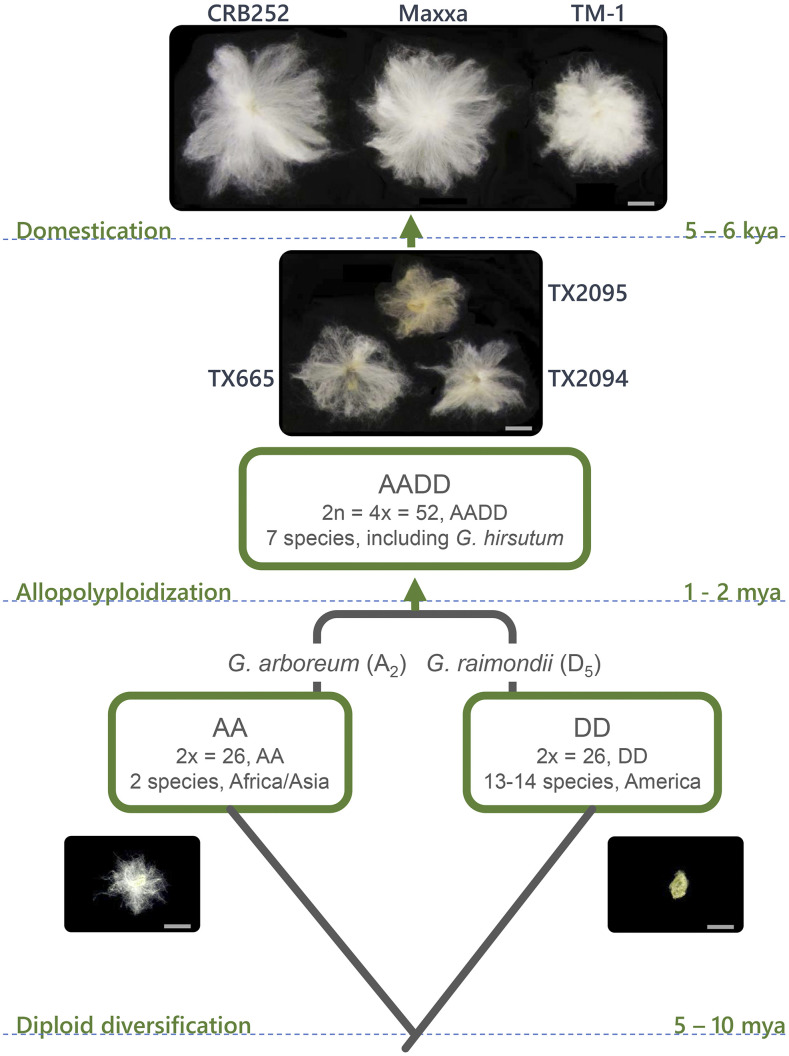
An abbreviated evolutionary history of cotton, including the model diploid progenitor species and estimated times for divergence and domestication. Branch lengths are not drawn to scale. Wild and domesticated *G. hirsutum* seeds are shown for each species listed, illustrating the effects of divergence and domestication on the epidermal seed trichomes (“fibers”). A 10mm scale bar is shown with each seed. Each of the six accessions examined in this study are represented by a single seed with attached fibers. TX2094, TX2095, and TX665 represent wild accessions, whereas CRB252, Maxxa, and TM-1 represent the improved, domesticated cultivars.

Cotton fibers are single-celled seed trichomes that differentiate from the ovular epidermis as early as three days before anthesis (Haigler *et al.* 2012). Fiber cells first elongate rapidly through the synthesis of a thin primary cell wall, which is followed by secondary cell wall thickening. During this transitional stage, around 15 to 20 days post-anthesis (dpa), an intermediary cell wall “winding” layer is deposited, which substantially increases fiber strength. This secondary cell wall, composed of 98–99% cellulose in domesticated cotton, continues to thicken until ∼40 dpa (Applequist *et al.* 2001; Haigler *et al.* 2012). During the last stage of fiber development, mature fibers dehydrate, form spiral twists, and the capsules (colloquially “bolls”) dehisce. This developmental program requires the regulation and coordination of hundreds to thousands of genes involved in cell wall and cytoskeletal formation (Szymanski and Cosgrove 2009; Haigler *et al.* 2012; Yoo and Wendel 2014; Fang 2018).

Although cotton fibers are single-cells primarily composed of cellulose, a high proportion of the ∼72,000 genes in the genome are expressed at some point during fiber development (Hovav *et al.* 2008c; Yoo and Wendel 2014; Tuttle *et al.* 2015). Previous microarray and RNA-seq studies have shown that the fiber transcriptomes vary dramatically between wild and domesticated *G. hirsutum* (Rapp *et al.* 2010; Yoo and Wendel 2014; Bao *et al.* 2019). Bao *et al.* (2019) showed that 15% of the genes have experienced some form of regulatory alternation between a pair of wild and domesticated *G. hirsutum* accessions (TX2094 and Maxxa, respectively) in 10 and 20 dpa fibers, developmental time points that represent primary wall synthesis and the transition to secondary wall synthesis, respectively. They and others (*e.g.*, Applequist *et al.* 2001; Yoo and Wendel 2014) suggested that the transcriptomes of modern elite lines have been reprogrammed so that the duration of fiber elongation is lengthened and that resources from stress response pathways have been reallocated toward enhanced fiber growth. While several classes of domestication-related genes have been identified [*e.g.*, master transcription factors (Shan *et al.* 2014); cellulose synthase (Rapp *et al.* 2010; Yoo and Wendel 2014), profilin (Bao *et al.* 2011), reactive oxygen species regulation-related enzymes (ROS) (Hovav *et al.* 2008b)], the underlying mechanisms and key components targeted by human-mediated selection remain elusive.

In addition to domestication, polyploidy itself also has had pervasive effects on gene expression underlying fiber development. *G. hirsutum* and six other tetraploid species (Krapovickas and Seijo 2008; Grover *et al.* 2015b, 2015a; Gallagher *et al.* 2017) represent the descendants of a single allopolyploidization event *ca*. 1-2 million years ago between an African, A-genome diploid species and an American, D-genome diploid (Wendel and Grover 2015). This hybridization and subsequent genome doubling reunited genomes that evolved independently for 5-10 million years on separate continents and created genome-wide gene duplicates (homoeologs). While these homoeologs exist in a shared *trans* regulatory environment, they may have divergent *cis* regulatory controls (Wendel and Grover 2015; Bao *et al.* 2019). The interactions among these newly reunited genes may contribute, in part, to the phenomenon known as “genomic shock”, whose myriad possible effects are summarized elsewhere (Jackson and Chen 2010; Grover *et al.* 2012; Yoo *et al.* 2014; Hu and Wendel 2019).

Adding to the evolutionary interest surrounding cotton fibers, only one parent of the polyploid species, the maternal A-genome progenitor, possesses long spinnable fiber. Accordingly, one might expect that the A-derived subgenome of the domesticated polyploids would carry most targets for selection of fiber-related traits. Contrary to this expectation, however, there is abundant evidence of D-genome recruitment into the developmental program of domesticated allopolyploid cotton fiber. Numerous QTL studies have found fiber-related loci in the D-subgenome of polyploidy cotton (Jiang *et al.* 1998; Lacape *et al.* 2005, 2010; Ulloa *et al.* 2005; Han *et al.* 2006; Rong *et al.* 2007; Qin *et al.* 2008; Said *et al.* 2013, 2015; Grover *et al.* 2019). Initial assessments of gene expression mirrored these observations, with 20% of homoeolog pairs exhibiting biased homoeolog expression in cotton fiber favoring homoeologs from the non-fiber (D genome) producing parent (Hovav *et al.* 2008b). Subsequent research has found a general balance between A- and D-subgenome expression but with considerable variation among genes with respect to the direction (A or D) of homoeolog bias (Yoo and Wendel 2014; Zhang *et al.* 2015; Fang *et al.* 2017b). The importance and contribution of the D-subgenome to allopolyploid cotton has recently been reiterated using whole genome resequencing screens for signatures of selection (Fang *et al.* 2017a, 2017b, 2017c; Wang *et al.* 2017). Collectively, the foregoing studies support the notion that polyploidy-related expression alterations underlie the transgressive and enhanced properties of fiber from allopolyploid relative to diploid cotton.

Whereas differential gene expression (DGE) is commonly used to evaluate transcriptomic changes among species and genotypes, coexpression analyses, including differential correlation (DC) and network construction, uncover how expression among genes is coordinated and how these coordinated relationships are evolutionarily altered by either natural or human-mediated selection. As demonstrated in tomato, wheat, maize, and cotton (Swanson-Wagner *et al.* 2012; Ichihashi *et al.* 2014; Pfeifer *et al.* 2014; Hu *et al.* 2016), coexpression networks provide a framework for testing preservation of coexpression patterns between wild and domesticated accessions or between diploid and polyploid species (Gallagher *et al.* 2016), while also highlighting coordinated changes among closely connected genes. Here we characterize fiber gene expression in wild and domesticated *G. hirsutum* across four time points representing key stages in fiber development. We examine the effects of polyploidy and domestication on gene coexpression, confirming previous reports of dramatic alterations in gene expression dynamics under domestication. While comparisons between homoeologous coexpression networks (*i.e.*, A- and D- subgenome networks) show general conservation in homoeologous network structure, comparisons between wild and domesticated networks show a distinct lack of conservation and a high level of differentially correlated genes. These results suggest a general preservation of duplicate gene function in polyploid *Gossypium* and highlight tighter co-regulation of fiber development genes following domestication, as observed for domesticated cottonseed (Hu *et al.* 2016).

## Materials and Methods

### Plant materials, mRNA sequencing and mapping

We selected three wild and three domesticated accessions of *G. hirsutum* to represent the wild to domestication transformation (Figure 1). These accessions have previously been shown to span the known genetic diversity of wild and domesticated cotton (Brubaker and Wendel 1994; Wendel *et al.* 1992; Grover *et al.* 2017; Yoo and Wendel 2014). Plants were grown in a common greenhouse environment in the Pohl Conservatory at Iowa State University. Cotton ovules were collected at 5, 10, 15, and 20 days post anthesis (dpa) and immediately frozen in liquid nitrogen for fiber RNA extraction. Briefly, frozen ovules were vortexed with glass beads to shear the fiber cells, and total RNA was extracted using the Sigma-Aldrich Spectrum Plant Total RNA kit (Sigma-Aldrich, St. Louis, MO) followed by purification as in Hovav *et al.* (2008c). Illumina RNA-seq libraries were constructed and subsequently sequenced as single-end 100-nt reads on the Illumina HiSeq2500 at the DNA Facility of Iowa State University (http://dna.biotech.iastate.edu/). A total of twenty-four RNA-seq libraries were generated with an average of 30 million reads per library.

RNA-seq reads were mapped onto the diploid reference genome sequence of D-genome diploid *G. raimondii* (Paterson *et al.* 2012) using the single nucleotide polymorphism (SNP)-tolerant mapping option of GSNAP (Wu and Nacu 2010) and a homoeolog-specific SNP database that distinguishes the A- and D- subgenomes in allopolyploid cotton (Page *et al.* 2014). Mapped reads containing the diagnostic SNPs were partitioned to estimate A- and D-subgenome homoeolog-specific expression by PolyCat v. 1.3 (Page *et al.* 2013, 2014). Read count data were generated via samtools (Li *et al.* 2009) and HTseq-count (Anders *et al.* 2015) for (1) the combined expression of both A- and D- homoeologous genes, and (2) the expression of individual homoeologs based only on subgenome specific reads. These two datasets are hereafter referred to as “joint” and “homoeologous”, respectively.

### Differential gene expression analysis

Using the R package DEseq2 (Love *et al.* 2014), a multifactor design of ∼ *domestication* + *development* + *domestication:development* was applied to both the joint and homoeologous datasets, together with pairwise comparisons of appropriate contrasts with respect to developmental stage and domestication status. Read counts greater than an average of 1 across all raw samples were considered expressed. The significant effect of *domestication:development* interaction term was determined by contrasting the full model against a reduced model of ∼ *domestication* + *development* with the likelihood ratio test (LRT) built in DESeq2. Significant statistical results were considered at a Benjamini-Hochberg adjusted *P*-value < 0.05 (Benjamini and Hochberg 1995). Functional enrichment analysis was performed using the topGO package in R 3.3.1 (Alexa and Rahnenfuhrer 2016; R Core Team 2017) with Fisher’s exact test. Gene ontology (GO) annotations for the *G. raimondii* reference genes was downloaded from CottonGen (Paterson *et al.* 2012; Yu *et al.* 2014).

Homoeolog expression bias was calculated in a similar fashion. Using the R package DEseq2 (Love *et al.* 2014), the multifactor design was updated to include “subgenome” as a factor (*i.e.*, ∼ subgenome + domestication + development). The data set was limited to gene pairs that showed an average expression of 1 read in at least half of the partitioned libraries. Differential expression between homoeologous copies was calculated by contrasting the A and D subgenome. Statistically significant homoeolog bias (within module or in total) was considered at a chi-square P-value <0.05.

### Weighted gene coexpression network construction

Gene coexpression networks were constructed using the R package WGCNA (Langfelder and Horvath 2008). After removing genes with zero expression or without variation across samples, a total of 29,706 homoeologous gene pairs were used to construct the joint networks, and 50,996 individual homoeologs (25,474 At and 25,522 Dt) were used to constructed the homoeologous networks. Read counts across different RNA-seq libraries were first normalized using the *rlog* function of DESeq2 (Love *et al.* 2014), and then subjected to automatic network construction using the WGCNA function *blockwiseModules* with default settings. Briefly, Pearson correlations were calculated between each pair of genes, and the resulting correlation matrix was raised to a default power of β = 12 to generate an adjacency matrix representing the connection strengths among genes. Adequate fit to the scale-free topology of the biological network was verified for each adjacency matrix (with the fit index above 0.8, or the highest fit index achieved). Next, the topological overlap matrix (TOM) was calculated to measure network interconnectedness for each pair of genes relative to all the other genes within the network. By performing average linkage hierarchical clustering with a dynamic tree cutting algorithm on 1 – TOM (the measure of topological overlap dissimilarity), highly interconnected genes were grouped together into coexpression modules, representing subnetwork structure and organization.

The module eigengene (ME), whose expression represents the members of a given coexpression module, was calculated as the first principal component of the scaled expression profiles of all module gene members. As previously done by Hu *et al.* (2016), module expression levels of member genes were summarized by their module eigengene value in correlation with the sample conditions (here, 2 cultivation conditions × 4 developmental stages = 8 conditions). Module eigengene-based connectivity (kME), also known as module membership, was calculated for each gene by the Pearson correlation between the gene expression profile and its corresponding ME. To determine whether a set of genes (*e.g.*, a list of differentially expressed genes or genes belonging to a Gene Ontology functional category) was significantly enriched within a specific module, ranked lists of module kME were subjected to gene set enrichment analysis (GSEA) using the *Preranked* function (Subramanian *et al.* 2005). That is, for a given gene set (*i.e.*, genes corresponding to a certain GO term or a differential expression category), the distribution of its gene members were examined in a full network gene list ranked by kME, thereby calculating an enrichment score to reflect the degree to which this gene set is overrepresented at the top of the entire ranked list. Compared to overlap based enrichment tests such as Fisher’s exact test, this approach is more robust to the threshold parameters used for defining WGCNA modules. The results of GSEA Preranked GO enrichment were visually summarized using REVIGO with an allowed GO term similarity of 0.7 and the *Arabidopsis thaliana* GO term size database (Supek *et al.* 2011).

### Conservation and divergence of gene coexpression networks

Network preservation tests were performed as previously described (Hu *et al.* 2016). To assess how well the intra-modular structure of a reference network is preserved in a test network, the WGCNA function *modulePreservation* calculates two types of preservation statistics, *i.e.*, *Z*_summary_ and medianRank scores, for each module (Langfelder and Horvath 2008). The medianRank is a composite module preservation statistic calculated to compare relative preservation among modules, such that lower medianRank score of a module indicates higher preservation relative to other modules. *Z*_summary_ is derived from the Z statictic; modules with *Z*_summary_ >10 are interpreted as strongly preserved, whereas *Z*_summary_ between 2 and 10 indicates weak to moderate preservation, and *Z*_summary_ < 2 indicates no preservation (Langfelder *et al*. 2011). Given that the *Z*_summary_ statistic is sensitive to module size, while medianRank is not, both statistics were considered collectively for inference of network modular structure preservation. This test was performed for (1) each homoeologous network *vs.* the joint gene expression modules and (2) for the domesticated homoeologous network *vs.* the homoeologous network from the wild accessions.

### Differential coexpression analysis

Differential coexpression (DC) analysis was performed by calculating the Pearson correlation coefficients for all gene pairs, followed by comparisons of corresponding gene pairs between wild and domesticated datasets, as previously conducted for cottonseed (Hu *et al.*, 2016). Differential correlation was evaluated based on Fisher’s z-test using the R package DiffCorr with a local FDR < 0.05 (Fukushima 2013). Differentially coexpressed genes were identified if the number of DC gene pairs was significantly higher than expected. That is, for a gene identified with *k* DC pairs among all gene pairs *n*, the probability *P* of this gene to be significantly coexpressed follows the binomial distribution model. The resulting *P* values were further corrected by the Benjamini-Hochberg method (Benjamini and Hochberg 1995) for multiple testing to detect significant DC genes (adjusted *P* < 0.05).

### Data availability

The raw sequencing data are available in the NCBI short read archive (SRA) under PRJNA530448 and on Dryad (DOI: http://dx.doi.org/10.5061/dryad.256hn). Supplemental material in support of this work can be found on figshare and includes: Fig. S1, RNA-seq library PCA by timepoint; Fig. S2, REVIGO plots summarizing homoeologous gene coexpression network module GO term enrichment; Fig. S3, patterns of module eigengene expression for each homoeologous gene coexpression network module; Fig. S4, gene membership correspondence between the wild and domesticated gene coexpression networks; Table S1, RNA-seq library read mapping and counts summary; Table S2, GO enrichment results for differentially expressed genes; Table S3, basic statistics for the joint and homoeologous gene coexpression networks; Table S4, GO enrichment results for the homoeologous gene coexpression network modules; Table S5, basic statistics for the wild and domesticated gene coexpression networks; Table S6, GO enrichment results for the differentially coexpressed genes; Table S7, genes showing both differential expression and differential coexpression; Table S8, genes showing both differential expression and differential coexpression related to cell wall biosynthesis and the cytoskeleton. Custom R scripts of the analysis performed in this paper are available at https://github.com/Wendellab/AD1FiberDom. Supplemental material available at figshare: https://doi.org/10.25387/g3.12268739.

## Results

### Transcriptome dynamics accompanying fiber development and domestication

**Figure 2:**
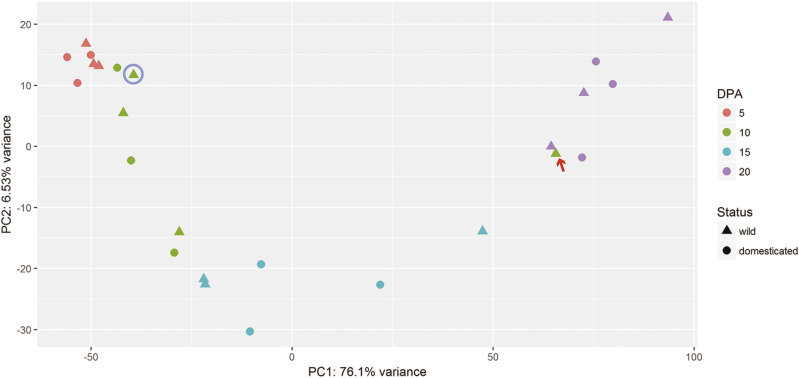
PCA of RNA-seq libraries. PC1, which accounts for more than three quarters of the variance (76.1%), is closely related to developmental stage, which ranges from 5 to 20 days post-anthesis (dpa; key to the right). The red arrow denotes a wild 10 dpa sample that grouped with the 20 dpa samples; this library was excluded from the analyses and replaced by a previously sequenced *G. hirsutum* var. *yucatanense* (TX2094) sample (circled).

Here, we compared gene expression in fiber for three wild and three domesticated accessions of *G. hirsutum* across four developmental time points. These accessions have previously been shown to encompass the genetic diversity within the wild and domesticated *G. hirsutum* gene pools (Wendel *et al.* 1992; Brubaker and Wendel 1994; Grover *et al.* 2017; Yoo and Wendel 2014). These time points represent primary fiber cell elongation (5 and 10 dpa) and the transition to secondary cell wall synthesis (15 and 20 dpa; Applequist *et al.* 2001; Rapp *et al.* 2010; Haigler *et al.* 2012). A total of 24 RNA-seq libraries were generated with an average of 30 million reads per library (Table S1). About 83.6% of raw reads were mapped to the SNP-tolerant *G. raimondii* reference genome (Paterson *et al.* 2012), with approximately equal proportions of homoeolog-specific reads assigned to the A- and D- subgenomes (Table S1). Principal components analysis (PCA) of the fiber transcriptome profiles revealed that the first component accounts for 76.1% of the total variance and mainly clusters fiber samples by developmental stage (Figure 2); there was no apparent clustering based on domestication. Clustering based on domestication was observed, however, when we performed PCA on each individual timepoints (Fig. S1). We noted that a single 10 dpa library of *G. hirsutum* var. *yucatanense* clustered with the remaining 20 dpa samples (Figure 2; red arrow), indicating a potentially mislabeled library; therefore, we replaced this sample with a previously sequenced RNA-seq library of 10 dpa *G. hirsutum* var. *yucatanense* fibers (SRX062250; Figure 2, blue circle) for all subsequent analyses.

Consistent with previous reports, (Hovav *et al.* 2008c; Yoo and Wendel 2014; Fang *et al.* 2017b), the majority of all cotton genes (including homoeologs) were expressed during fiber development. A total of 29,706 homoeologous gene pairs were jointly expressed during these timepoints, accounting for 79.2% of the 37,505 reference gene models in the diploid *G. raimondii* genome. When considering the homoeologs individually, expression is slightly lower; *i.e.*, 68.0% of homoeologs were expressed in developing fibers. The number of homoeologs expressed from each of the two subgenomes was approximately evenly distributed, 25,474 and 25,522 in the A- and D- subgenomes, respectively.

In order to disentangle the fiber transcriptomic changes due to the effects of *domestication*, *development*, or their complex interactions, we conducted differential gene expression (DGE) analyses by employing a multivariate design of “∼ *domestication* + *development* + *domestication:development*”s (Table 1, top) in addition to univariate, pairwise comparisons (Table 1, bottom). By blocking the accessions selected specifically so that they represented the endpoints “wild” *vs.* “domesticated”, thereby treating accessions as pseudo-biological replicates, we are able to better identify regulatory evolution that has accompanied the domestication process (Yoo and Wendel 2014). Common to both wild and domesticated accessions, significant effects of *development* were found for 29,672 individual homoeologs (58.2% of those expressed). Pairwise comparisons of adjacent developmental stages (Table 1) showed that the largest DGE changes occur between 15 and 20 dpa, which represents the transition from primary elongation to secondary wall synthesis (Haigler *et al.* 2012). The lowest amount of DGE was found between 10 and 15 dpa fibers. Gene Ontology (GO) term enrichment analysis suggests that these DGE genes with developmental effects are primarily involved in metabolism, biosynthesis, and transport (adjusted *P* < 0.05; Table S2). During primary wall synthesis (here, 5 and 10 dpa), regulation-related GO terms (adjusted *P* < 0.05; Table S2) were significantly down-regulated for both wild and domesticated fiber. Toward the end of elongation (*i.e.*, 10 to 15 dpa), fatty acid and lipid biosynthesis and metabolism related GO terms were enriched for genes upregulated only in the domesticated accessions, (adjusted *P* < 0.05;. Table S2), which may suggest that lipids, as components of cell membranes and vesicles, are trafficking necessary molecular components to the expanding fiber cell wall. On the contrary, genes upregulated in wild fibers at 10 to 15 dpa were enriched for cellulose biosynthesis and metabolism, suggesting an earlier initiation of secondary cell wall biosynthesis, as previously shown (Table S2; Butterworth *et al.* 2009). DGE was highest during the transition to secondary cell wall biosynthesis (between 15 and 20 dpa), where a staggering number of GO categories were enriched (Table S2); these include various biosynthesis, transport, and molecule modification terms, including those related to cell wall biosynthesis.

**Table 1:** Differential expression analysis of homoeologous fiber transcriptomes

multivariate, LRT tests			
*development*		29,671	
*domestication*		10,218	
interactive effect of *domestication* over *developmental* stages			
10 v 5 dpa		11	
15 v 10 dpa		15	
20 v 15 dpa		18	
overall		85	

univariate, pairwise comparisons			
			
		**log2 Fold Change > 0**	**log2 Fold Change < 0**
Wild *vs.* Domesticated at:			
5 dpa		517	288
10 dpa		563	291
15 dpa		419	273
20 dpa		762	745
Wild, between adjacent time points:			
10 v 5 dpa		1052	214
15 v 10 dpa		754	360
20 v 15 dpa		2531	2918
Domesticated, between adjacent time points:			
10 v 5 dpa		1225	407
15 v 10 dpa		695	571
20 v 15 dpa		3983	3322

Following the effect of *development*, a smaller number of homoeologs (10,218; 20% of expressed genes) were identified to show significant *domestication* effect, regardless of dynamic changes during fiber development. Here, direct comparisons between wild and domesticated accessions at each developmental stage revealed the most DGE at the latest stage examined, *i.e.*, 20 dpa, whereas the least DGE was observed at the beginning of the transition stage (15 dpa; Table 1). These *domestication* DGE genes were found to be associated with a large number of GO categories, including localization, transport, and detection of stimuli (adjusted *P* < 0.05; Table S2). For pairwise comparisons, the highest number of enriched GO terms was observed at 20 dpa, including small molecule biosynthesis and metabolism-related processes, which were upregulated in wild fiber (adjusted *P* < 0.05; Table S2).

Only 85 DGE genes exhibited a significant interaction effect of *domestication* over *developmental stages* (Table 1). DGE was greatest between 15 and 20 dpa; however, given the small number of detected genes, no GO categories were enriched for this interaction comparison.

### Highly conserved modular organization between A- and D- homoelogous networks in allopolyploid cotton fiber

Coexpression network analysis has an additional dimension in polyploid species where the duplicated nature of the genome allows individual homoeologs to retain their original function, to evolve independently (possibly acquiring new or partitioned functions), and to interact in a variety of adaptive, maladaptive, and neutral ways. In considering polyploid coexpression networks, one approach is to treat all homoeologous pairs just as one would treat alleles of a single gene, regardless of parental origin. Alternatively, each homoeologous network (comprising homoeologs of the same parental origin) might be considered independently to reveal how networks respond to polyploidization. While the assumption that homoeologous pairs are equivalently co-regulated will be violated for any number of genes, construction of a coexpression network from the summed expression of all homoeologs into a single “gene pair” (*i.e.*, treating them as “alleles”) provides a null model (referred to as the “joint network” hereafter; Table 2) for comparison to individual, homoeologous gene networks (referred to as the “homoeologous network” hereafter, Table 2). Here, such comparisons were conducted to detect differences that may be relevant to fiber development and domestication, and to explore network-level responses to genome doubling.

**Table 2:** Coexpression networks constructed in this study

Network Name	Accessions Included	Basis of Coexpression Relationships
Joint	All accessions	Sum of Homoeologous Gene Pair Expression
Homoeologous	All accessions	Individual Gene Expression
Wild	TX665, TX2094, TX2095	Individual Gene Expression
Domesticated	CRB252, Maxxa, TM-1	Individual Gene Expression

**Figure 3:**
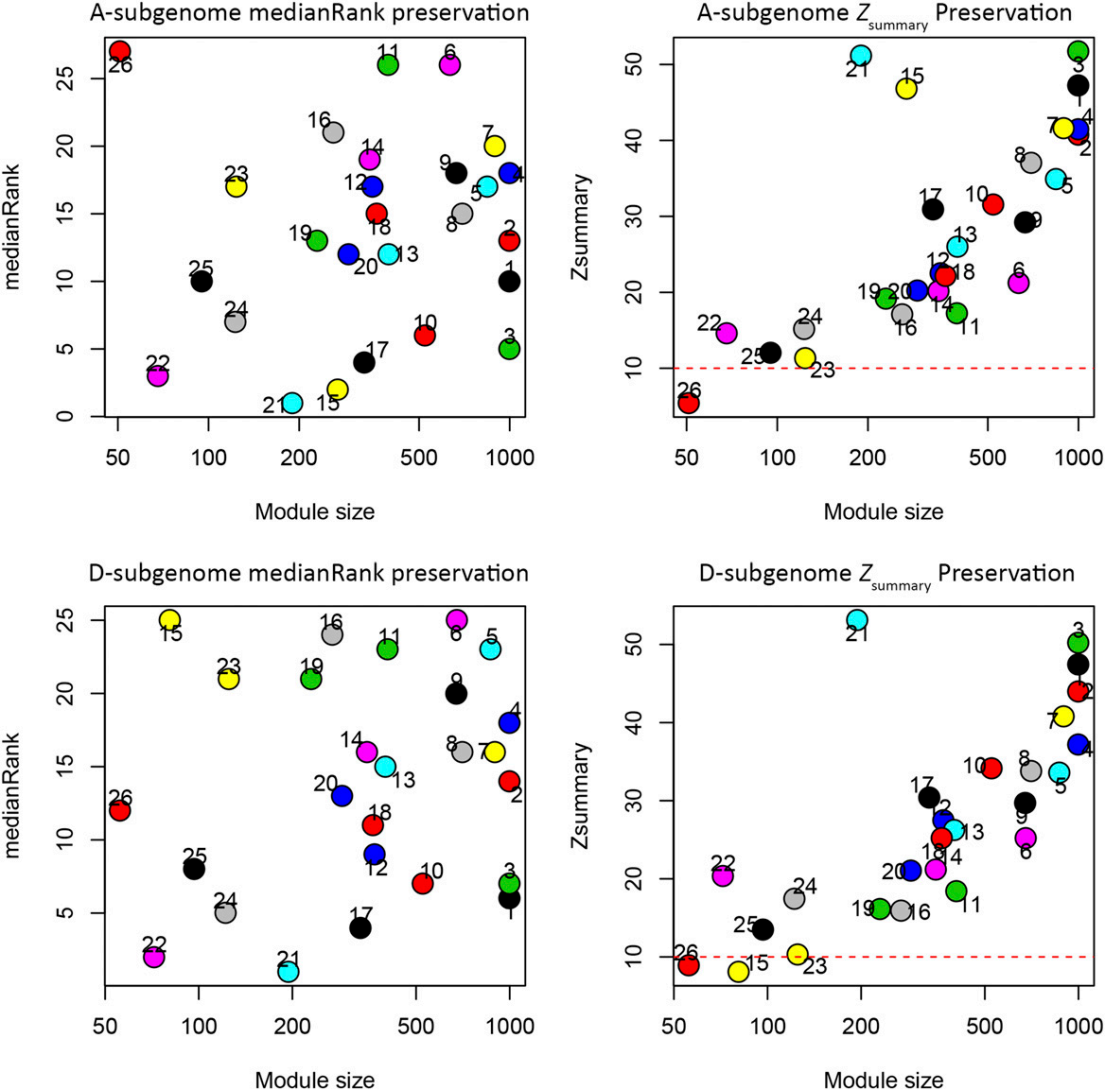
Module preservation of joint gene coexpression network topology in A- and D-subgenomes. The top two graphs show joint module preservation in the A-subgenome fiber expression data, while the bottom two graphs show the joint module preservation in the D-subgenome fiber expression data. Module numbers are shown on graphs; the same numbers correspond to the same joint modules. Red dashed line marks the preservation threshold at *Z*_summary_ = 10. For medianRank, a lower score indicates higher preservation relative to higher scores.

Construction of the joint network resulted in partitioning the 29,706 gene pairs into 26 modules (*i.e.*, clusters of highly co-expressed genes; Table S3). Using this joint network as reference, we asked whether its modular organization was separately preserved by both the A- and D- homoeologous gene expression datasets. When the homoeologous gene expression data (*i.e.*, from 25,474 A- and 25,522 D-homoeologs) was fit to the modular organization of the gene-pair (*i.e.*, joint) network, nearly all modules exhibited high preservation scores for both the A- and D-homoeologous networks (*Z*_summary_ > 10, see Methods; Figure 3). This indicates general preservation of gene-pair coexpression relationships by both the A- and D- subgenomes. Notably, a lack of preservation was observed for modules 15 and 26 in either one or both of the homoeologous networks (*Z*_summary_ < 10 and high medianRank scores; Figure 3). For module 26, this may be due to a lack of statistical power, given it contains the smallest number of genes (n = 72). For module 15, however, poor preservation according to both Z_summary_ score and the medianRank score is only observed in the D-homoeologous network, potentially representing expression and/or functional divergence for genes contained in that module.

In addition to evaluating the topological similarity between each individual homoeologous network relative to the joint network, we also assessed the expression contribution of each homoeolog (*i.e.*, homoeolog expression bias) across each module in the joint network (Table 3). Of the 16,273 gene pairs that exhibit homoeolog expression bias (based on DGE, see methods), significantly more pairs display higher expression of the D-homoeolog than the A-homoeolog (8521 *vs.* 7752; Chi-squared test *P* < 0.05; Table 3). At the module level, 16 modules contained balanced numbers of A- and D- biased expression, while 9 modules exhibited D-biased expression (not including Module 0, which contains unassigned genes) and only one module exhibited a strong bias toward higher expression of A-homoeologs (*i.e.*, module 15, which contains 216 A-biased *vs.* 24 D-biased gene pairs; Table 3). This was the same A-biased module that showed asymmetrical preservation of the joint topology between the A- and D-homoeologous expression datasets. Considering that almost all other modules in the joint network were well preserved by homoeologous networks, the observed general and module-level D-homoeolog biases do not appear to significantly alter gene co-expression relationships.

**Table 3:** Homoeolog expression bias by module for the homoeolog-pair network

	Gene Number			chi-square	Module level bias
Module ID	total	A-biased	D-biased	*P* value	(*P* < 0.05)
0	615	280	335	0.03	D-bias
1	3747	1800	1947	0.02	D-bias
2	4591	2180	2411	0.00	D-bias
3	1072	498	574	0.02	D-bias
4	754	360	394	0.22	
5	665	339	326	0.61	
6	567	251	316	0.01	D-bias
7	497	220	277	0.01	D-bias
8	497	242	255	0.56	
9	481	229	252	0.29	
10	302	118	184	0.00	D-bias
11	252	117	135	0.26	
12	288	128	160	0.06	
13	251	124	127	0.85	
14	261	118	143	0.12	
15	240	216	24	0.00	A-bias
16	186	80	106	0.06	
17	122	61	61	1.00	
18	240	101	139	0.01	D-bias
19	132	61	71	0.38	
20	185	87	98	0.42	
21	33	13	20	0.22	
22	34	19	15	0.49	
23	77	39	38	0.91	
24	71	27	44	0.04	D-bias
25	65	24	41	0.03	D-bias
26	48	20	28	0.25	
**Sum**	16273	7752	8521	0	Unbalanced

### The majority of homoeologous gene pairs are in separate modules in the polyploid network

We next constructed the homoeologous coexpression network based on the expression of individual homoeologs. A total of 50,996 homoeologs expressed during fiber development (see above) were clustered into 52 coexpressed modules containing between 38 and 9,314 genes (Table S3). Notably, this doubles the number of joint network modules (52 *vs.* 26), indicating that, while the general network topology is largely preserved, coexpression relationships within each subgenome are distinct enough to generate separate modules. In support of this hypothesis, only one fifth of all paired homoeologs (*i.e.*, 7,561 out of 37,505 homoeologous gene pairs) were placed into the same module. This implies that the coexpression divergence between homoeologous genes mainly occurred at the intermodular level, whereas the intramodular relationships are most likely preserved.

Among these 52 modules, 28 exhibited expression profiles that were significantly associated with developmental stage and/or domestication status (ANOVA, *P* < 0.05; Fig. S3). DGE genes exhibiting significant *development* and/or *domestication* effects were enriched in 22 modules (42%; GSEA adjusted *P* < 0.05). Among those modules, 20 exhibited enrichment for *development* DGE, with 14 modules also enriched for *development*:*domestication* interaction DGE (Table S3). Enrichment for the *domestication* effects was found for 11 modules, which usually also were enriched for *development* (8 modules) and *development*:*domestication* (10 modules) effects (GSEA adjusted *P* < 0.5; Table S3).

**Figure 4:**
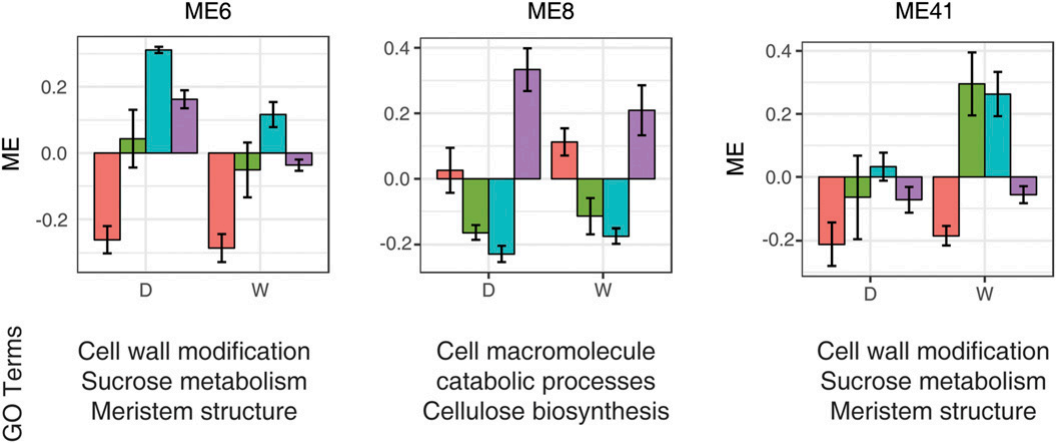
Module eigengene expression of select modules from the homoeologous fiber network. For each module, the barplot presents the module eigengene expression levels centered by means across developmental timepoints for wild and domesticated fiber. Error bars represent the standard errors among three accessions for each genome group at each developmental stage. Developmental stages: red,5 dpa; green, 10 dpa; blue, 15 dpa; purple, 20 dpa.

Each module was functionally annotated by enriched GO terms, where several modules were identified with relevance to key biological processes of fiber development (Table S4, Fig. S2). Module 6, for example, was enriched for cell wall modification, sucrose metabolic and biosynthetic process, and regulation of meristem structural organization (GSEA, adjusted *P* < 0.05; Table S4) and showed higher expression at 15 and 20 dpa (Figure 4). Module 8 also showed enrichment for a large number of biological processes, including cellulose biosynthetic process and cell wall macromolecule catabolic process (GSEA, adjusted *P* < 0.05; Table S4); this module showed low expression at 10 and 15 dpa, but spiked at 20 dpa, when the secondary cell wall is forming (Figure 4). Module 41, enriched for cell wall modification, regulation of meristem structural organization, and sucrose biosynthetic processes (GSEA, adjusted *P* < 0.05; Table S4), showed higher expression at 10 dpa and 15 dpa in wild fiber, but much lower expression in domesticated fiber (Figure 4). Other modules also showed enrichment for GO terms related to the cell wall (module 9), meristem structure and development (modules 7,12,17), cellulose (modules 28,33,37), and sucrose (modules 1, 9, 17, 23, 37, 44, 46); however these GO terms only represent a subset of the enrichment observed (Table S4, Fig. S2).

### Domestication has substantially altered the fiber network topology

Previous work on the cotton seed transcriptome revealed the extensive effects of domestication on the coexpression relationships among genes and homoeologs (Hu *et al.* 2016), a key conclusion being that interconnectivities became more tightly regulated in domesticated cotton. Here, we observed a similar effect in the fiber transcriptome, by comparing the individual coexpression networks each constructed from the wild and domesticated homoeolog expression datasets (Table 2). Over twice as many gene modules were recovered from the wild *vs.* domesticated network (107 *vs.* 47 modules, respectively; Table S5), suggesting tighter coregulation of genes in domesticated fiber. While a similar number of modules were found significantly associated with fiber development in both networks (10 and 9, respectively; ANOVA, *P* < 0.05, Table S5), these modules do not contain similar sets of genes according to the marginal correspondence analysis (Fig. S4, bolded modules). Instead, over 10 times more genes were clustered in these development-related modules in the domesticated network (33,037 genes) than those in the wild network (2,426 genes). It appears that several modules not significantly related to fiber development in wild cotton were recruited and became co-regulated during domestication.

**Figure 5:**
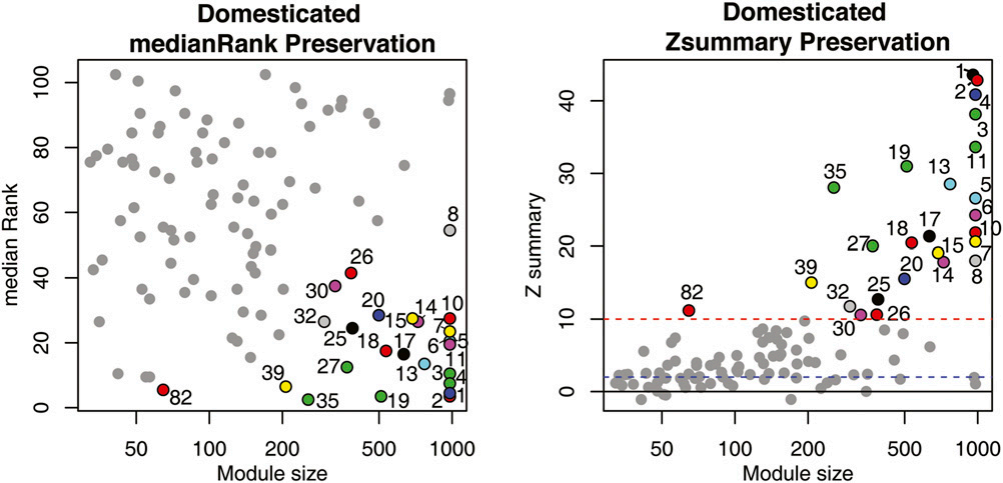
Preservation of wild cotton fiber gene coexpression network modules in the domesticated cotton fiber gene expression data. Left panel: modules with lower medianRank scores are relatively more preserved than modules with higher medianRank scores. Right panel: modules above the red dashed line at *Z*_summary_ = 10 are considered well preserved. Modules with *Z*_summary_ < 10 were colored gray without labels for clarity. Blue dashed line, *Z*_summary_ = 2.

Module preservation tests (Figure 5) showed that of the 108 modules present in the wild cotton network, only 26 were strongly preserved when fitting the domesticated fiber gene expression data to the wild network topology (*Z*_summary_ > 10, low medianRank score), indicating a large reorganization of network relationships accompanying domestication. Moreover, preservation of the development-related modules is minimal, with only 3 strongly preserved in domesticated cotton. Taken together, these results show that domestication has condensed the coexpression network in cotton fibers, resulting in tighter and denser connections among genes, in a similar manner as for the cotton seed gene coexpression network (Hu *et al.* 2016).

In addition to the coexpression networks, differential correlation (DC) analysis was performed to directly contrast the gene-to-gene correlations between wild and domesticated transcriptomes. Nearly one third of all homoeologs (*i.e.*, 16,503 genes or 32.4%) exhibited significant DC change under domestication (adjusted *P* < 0.05; see Methods). GO enrichment analysis showed that these DC genes were enriched for a wide variety of Biological Processes GO Terms (*P* < 0.05, q < 0.05; Table S6; Alexa and Rahnenfuhrer 2016), including protein localization, protein transport, catabolic processes, and DNA metabolic processes. Among the DC genes, 10,026 overlapped with the list of DGE genes that showed differential expression between wild and domesticated cotton (*i.e.*, a *domestication* effect), containing roughly equivalent number of genes between subgenomes (4,941 A-homoeologs and 5,085 D-homoeologs; Table S7). This large list of overlapping DGE/DC genes also supports the observed substantial change in the cotton fiber transcriptome by domestication, and thus was also used to pinpoint the target domestication genes involved in key biological processes and functions in fiber.

### The effects of domestication on fiber cell wall synthesis

Changes in gene expression and co-regulation accompanying domestication represent the molecular consequences of intense, directional selection on the fiber phenotype. Obvious targets of selection include genes involved in specifying the composition and regulation of the cytoskeleton and fiber cell wall. Previous work has revealed some of the genes involved in cotton fiber synthesis, specifically those involved in cell wall biosynthesis and cytoskeletal activities (Haigler *et al.* 2009; Szymanski and Cosgrove 2009; Taylor-Teeples *et al.* 2015). While 150 genes related to the cytoskeleton and/or cell wall biosynthesis exhibited both significant DGE and DC across the developmental timepoints (Table S8), these were distributed among 11 (of 52) modules. These DGE/DC cell wall and cytoskeletal genes encode a diversity of proteins, including cellulose synthases, expansins, actins, tubulins, and ethylene response sensors, among others.

Examples of genes altered during domestication include several genes encoding proteins involved in generating the cell wall, *i.e.*, galacturonosyltransferase 3 (*GAUT3*), three glycosyl hydrolases, an exostosin gene, and a CESA gene. Only *GAUT3* was upregulated under domestication (at 5 and 10 dpa). The remaining five genes were all downregulated at various stages: the hydrolases at 5 dpa, the *CESA* gene at 15 dpa, and the exostosin gene at 10 and 15 dpa. This general trend of downregulation was also observed for genes encoding proteins involved in lignin biosynthesis, which is also relevant to cell wall synthesis, although the gene expression changes suggest a potential reduction in lignin content. Both O-methyltransferase and cytochrome P450 are involved in lignin synthesis and both were broadly downregulated (5/10/15 dpa and 20 dpa, respectively). A single laccase gene, which is involved in lignin degradation, shows a more complicated pattern, *i.e.*, upregulated in domesticated cotton at 5 dpa but downregulated at both 15 and 20 dpa. Finally, tubulin β, a microtubule component of the cytoskeletal framework, was also downregulated at 10 dpa. Interestingly, while one might expect other canonical cell-wall related genes to exhibit significant DE and DC, such as additional *CESA* homologs, actins/actin-regulating genes, other tubulins, and cytoskeletal motors, we did not observe any significant changes in the stages evaluated for *G. hirsutum*.

Also important in fiber development are genes related to regulation and/or cell signaling. We found several genes that have significant expression changes correlated with domestication. These include genes encoding three MYB transcription factors (*i.e.*, MYB3, 7, and 66), which were downregulated in domesticated cotton at 5 dpa (MYB3 and MYB7) and 20 dpa (MYB66) and a heavy metal transport protein (potentially involved in signaling), which was upregulated at 10 and 20 dpa. Also upregulated are homologs of *ERF1*, *ERS1*, and *ETR2*, which encode proteins functioning in ethylene response and regulation, and have been associated with cotton fiber elongation (Li *et al.* 2007).

Finally, we find a large number of genes (Table S7) encoding proteins that regulate reactive oxygen species (ROS), which are known to function in diverse cellular processes, including cell expansion (Cosgrove 2005), patterning (Gapper and Dolan 2006), and polar growth (Mori and Schroeder 2004). Previous research has shown upregulation of genes encoding ROS producing and regulating proteins in domesticated cotton fiber (Hovav *et al.* 2008a; Chaudhary *et al.* 2009). Accordingly, we see several peroxidase-encoding genes upregulated within different modules in domesticated fiber, in addition to two NADPH oxidoreductase-encoding genes.

While these and the above mentioned genes are not an exhaustive list of biologically relevant gene expression changes, they serve to underscore the complexity of the expression changes that have altered the fiber developmental program as a result of domestication. A complete list of fiber-related genes and their expression changes is in Table S8.

## Discussion

The suite of coordinated changes in gene expression that underlie polyploidy and domestication are of substantial interest from both evolutionary and agronomic standpoints. Decades of research have elucidated the myriad changes in gene expression that are stimulated during polyploidization and have highlighted the opportunities that a redundant genome might provide for evolutionary innovation (Osborn *et al.* 2003; Adams and Wendel 2005; Chen and Ni 2006; Chen 2007). Simultaneously, changes in gene expression under domestication have been studied in multiple species, either on a gene-by-gene basis (Hirano *et al.* 1998; Konishi *et al.* 2006; Cong *et al.* 2008) or genome-wide (Doebley *et al.* 2006; Hovav *et al.* 2008; Paran and van der Knaap 2007; Jin *et al.* 2008; Ramírez-González *et al.* 2018; Dong *et al.* 2019; Sauvage *et al.* 2017). Allopolyploid cotton provides the opportunity to evaluate the consequences of both processes, having been domesticated *ca*. 5000 years ago (Wendel *et al.* 2010).

Previous research in cotton has demonstrated that homoeologs commonly diverge in expression pattern, and possibly function (Chaudhary *et al.* 2009; Guan *et al.* 2014; Lv *et al.* 2016; Liu *et al.* 2019), and that domestication has dramatically altered the fiber transcriptome (Hovav *et al.* 2008b; Rapp *et al.* 2010; Yoo and Wendel 2014). We evaluate the gene coexpression network in developing fibers from both wild and domesticated representatives of *G. hirsutum* to characterize the patterns of expression evolution that have been caused by polyploidy and domestication.

### Coordinated expression changes of cell wall-related genes during domestication

Cotton fiber development is highly complex, involving myriad metabolic processes incorporating most of the genes in the genome. A much longer, stronger, finer, whiter fiber, one of the most obvious outcomes of directional selection under domestication, is likely controlled by those genes related to cell wall biosynthesis and the cytoskeleton. Here we find many of these genes are significantly differentially expressed *and* co-expressed across developmental timepoints and under domestication, suggesting a coordinated role in conferring the domesticated phenotype. For example, upregulation of *GAUT3* results in an increase in pectin (Sterling *et al.* 2006) and thereby cell wall flexibility (Stiff and Haigler 2012), while a concomitant downregulation of three glycosyl hydrolases reduces hydrolyzation of the glycosidic bonds found within the cell wall (Xu *et al.* 2004), thereby increasing stability of the primary cell wall during elongation. These exemplar genes represent distinct examples of a broader pattern of coordinated regulation among genes. We found large numbers of genes involved in reactive oxygen species (ROS) and ethylene biosynthesis/response that exhibited DE and DC, whose coordination between and among pathways affects cell wall loosening and regulation of elongation (Cosgrove 2005; Shi *et al.* 2006; Li *et al.* 2007; Hovav *et al.* 2008a; Qin *et al.* 2008; Chaudhary *et al.* 2009; Zhang *et al.* 2016), Likewise, we found coordination of multiple genes involved in regulating lignin production at the early stages of fiber development, which results in reduced lignin production in the early stages of fiber development and absence of lignin in the later stages, resulting in lighter and smoother fibers. Interestingly, although each fiber is composed primarily of cellulose, only a single CESA-encoding gene (*CESA8*-homolog) exhibited significant expression changes in our data and for only a single timepoint. Notably, no other cellulose synthase paralogs exhibited expression differences under domestication here despite the increased cellulose content of domesticated fiber, possibly indicating that regulation of cellulose synthase isoforms may not occur at the transcriptional level.

### Evolution of networks duplicated by polyploidy

Polyploidy, or whole genome duplication, is a recurrent and ongoing phenomenon that has influenced the evolutionary history of all plants (Jiao *et al.* 2011; Soltis *et al.* 2014a; Wendel 2015; Soltis *et al.* 2014b; One Thousand Plant Transcriptomes Ini...). The gene expression and regulatory consequences of polyploidization are many, and include extensive transcriptional rewiring as the independently evolved regulatory environments merge and interact (Ramírez-González *et al.* 2018; Yang *et al.* 2016; Yoo *et al.* 2014; Gallagher *et al.* 2016; Bao *et al.* 2019; Hu *et al.* 2016; Edger *et al.* 2019). Specifically, novel interactions between the *cis* and *trans* factors derived from each progenitor species have consequences for the polyploid expression environment, reverberating throughout the network (De Smet and Van de Peer 2012; Gallagher *et al.* 2016; Bao *et al.* 2019).

Previous work on cotton seed networks (Hu *et al.* 2016) demonstrated extensive rewiring of the seed oil network as a consequence of hybridization between the two independently evolved diploid progenitor species and subsequent genome doubling; however, this study was limited by aggregating the expression of homoeologous gene pairs into a single cumulative value. Here we extend this approach to compare the two co-resident homoeologous gene networks in allopolyploid cotton. We find that in general, each homoeologous network (the A- and D-subgenome networks) is largely similar to the joint network created by combining homoeologous gene expression (a *la* Hu *et al.* 2016), indicating a general preservation of network topology between the two homoeologous networks. Comparisons between the joint and homoeologous coexpression networks mirrors this observation of general conservation, as the homoeologous network contains the expected doubling of the number of modules (52 *vs.* 26 modules in the homoeologous *vs.* joint network, respectively).

In contrast to preservation at the module level, the homoeologous networks themselves were highly divergent in module membership. Comparisons between the A and D networks revealed that only a quarter of the expressed homoeologs were found in the same module. Together, these results indicate that the general topology of the fiber network is similar in the A- and D- homoeologous networks, but that coexpression differences among homoeologs are somewhat different. These observations are relevant to our understanding of polyploidy in general and to cotton in particular. With respect to the former, our results demonstrate a dimension of homoeologous coexpression evolution that has not previously been addressed, and which may comprise a common feature of allopolyploid genomes. Recent work in cotton has demonstrated complex interactions between independently evolved *cis* and *trans* acting factors (Bao *et al.* 2019), which become combined in a common *trans* environment with the onset of polyploidy. We speculate that the myriad novel interactions between regulatory sequences and *trans*-acting factors become superimposed on the pre-existing regulatory environments of the divergent diploid progenitors, magnifying expression and coexpression differences in the allopolyploid. Understanding how this new combinatorial complexity is shaped by the evolutionary process or in response to particular selective regimes remains a promising avenue for understanding polyploid evolution and the origin of new phenotypes.

With respect to cotton, our coexpression network results may offer insight into the transgressive, superior fibers of cultivated allopolyploid cotton relative to their diploid progenitors. The generalized conservation of coexpression modular structure for the A and D homoeologs suggests that even though the D-genome progenitor does not produce spinnable fiber, the underlying architecture of the developmental program for producing epidermal seed trichomes is conserved. This realization may lead to an enhanced understanding of both the superior fiber produced by polyploid cotton and previous observations of D-genome recruitment for fiber production during domestication (Jiang *et al.* 1998; Lacape *et al.* 2005, 2010; Ulloa *et al.* 2005; Han *et al.* 2006; Rong *et al.* 2007; Qin *et al.* 2008; Said *et al.* 2013, 2015; Grover *et al.* 2019). Our results thus direct attention to unraveling the evolutionary and regulatory differences responsible for the A *vs.* D expression and coexpression differences, and how these were altered by polyploidization and domestication.

The contribution of the D-genome to polyploid cotton fiber is reiterated by the generalized bias in expression toward this parent, both in overall gene expression and module expression. Although there are many studies demonstrating homoeolog bias in cotton (Hovav *et al.* 2008b; Yoo and Wendel 2014; Zhang *et al.* 2015), we provide a new perspective on this phenomenon here, relating homoeolog expression bias to coexpression module membership, domestication status, and developmental stage. Notably, our estimates of differential expression relative to domestication and development all show bias toward higher expression of the D-subgenome, but that differential coexpression analysis highlights the fact that an individual module may reverse this trend. Specifically, module 15 exhibits higher expression in the A-subgenome where it was also more strongly preserved. This module exhibits lower expression in domesticated cotton that is generally restricted toward the later developmental timepoints. These observations suggest that reducing expression of this module at specific timepoints may be important for the domesticated fiber phenotype, making those genes suitable targets for agronomic improvement via RNAi or other expression-reducing modification.

### Domestication recruits genes into more tightly regulated modules

Coexpression networks provide a useful summary of complex, multidimensional data, such as changes in transcriptional relationships among genes across developmental time and between accessions. Coexpression analysis of cotton seed domestication demonstrated, quite remarkably, that changes under domestication within a species were more extensive than natural evolutionary changes between species (Hu *et al.* 2016), and that domestication resulted in a more highly condensed, or tighter network. Similar to the latter, domestication has extensively rewired the cotton fiber network, resulting in fewer and more densely connected modules. While the number of modules was reduced by a little more than half in domesticated cotton, the number of genes associated with domestication-relevant modules increased 13-fold to nearly half of the genes in the allopolyploid genome. This results in nearly one-third of genes exhibiting evidence of differential correlation. These genes were largely recruited from wild modules that were not significantly associated with fiber development, and whose coordinated expression contributed to the domesticated fiber phenotype. This high level of change following cotton domestication is also concordant with the high number of QTL that are found in studies associated with wild and domesticated Upland cotton (Jiang *et al.* 1998; Lacape *et al.* 2005, 2010; Ulloa *et al.* 2005; Han *et al.* 2006; Rong *et al.* 2007; Hovav *et al.* 2008b; Qin *et al.* 2008; Said *et al.* 2013, 2015; Fang *et al.* 2017a, 2017c; Wang *et al.* 2017; Grover *et al.* 2019). While the results discussed here only represent only a small sampling of the wild and domesticated gene pools, the level of diversity within wild and domesticated cotton populations suggests that our results would be reiterated in an expanded sampling of accessions. Taken together, our results demonstrate the amazing complexity that underlies the domestication of cotton fiber.

Enrichment of highly connected intramodular hub genes can show functions that act together during fiber development. Gene set enrichment analysis (GSEA) was used to assess which modules were associated with various biological processes. We detected enrichment for genes within modules (Table S4, Fig. S2), with gene sets being enriched for processes such as cell wall biogenesis, fatty acid biosynthesis, flavonoid biosynthesis, and many others, underscoring the complexity of the cotton fiber transcriptome and its developmental dynamics. To some extent this is not an unexpected result, given the observation that the majority of the genes in the genome are expressed at some point during cotton fiber development; accordingly, we might expect complex coexpression relationships that involve or invoke the majority of cellular processes. We suggest that the modules described here as enriched for specific biological processes represent starting points for functional genomic studies targeting intramodular hub genes and for additional techniques designed to layer with transcriptomic coexpression data (*e.g.*, protein interaction networks, transcription factor binding networks).
